# Reconstructing sudden ambient temperature changes for forensic death time estimation using temperatures in two closed compartments: proof of concept

**DOI:** 10.1007/s00414-026-03767-4

**Published:** 2026-04-09

**Authors:** Jayant Shanmugam Subramaniam, Michael Hubig, Sebastian Schenkl, Holger Muggenthaler, Steffen Springer, Martin Weiser, Jakob Sudau, Faisal Shah, Gita Mall

**Affiliations:** 1 Forensic Science Institute at the University Hospital, Friedrich Schiller University Jena, Jena, Germany; 2 Zuse Institute Berlin, Berlin, Germany

**Keywords:** Temperature-based death time estimation, Sudden undocumented ambient temperature declines at crime scene, Reconstruction of former ambient temperature drop, Temperature trajectories in closed compartments at crime scene as input.

## Abstract

Ambient temperature T_A_ has a strong impact on temperature-based time since death estimation (TTDE). Frequently T_A_ is lowered instantaneously at some time t_0_ from a previous value T_A0_ to T_A1_ < T_A0_ by, e.g., opening a window or a door. We aim at reconstructing T_A0_ and t_0_. TTDE literature suggests temperature measurements in closed compartments such as cupboards or neighboring rooms, where T_A0_ could have been ‘preserved’ after t_0_. We aim to estimate t_0_ and T_A0_ from temperature measurements T_Z_(t) in closed compartments Z at times t > t_0_. We obtain promising results assuming Newtonian cooling for boxes filled with air, heaps of clothes, or books in two different experimental scenarios. Two different parameter estimators, (T_A0_^, t_0_^) based on four temperature measurements and (T_A0_*, t_0_*) for 4 N measurements were tested. A decline at time t_0_ from T_A0_ = 22.5 °C ↓ T_A1_ = 14 °C was reconstructed at t = t_0_ + 95 min with relative deviations ρt_0_^ = 27% and ρT_A0_^ = 19% relative to t - t_0_ and T_A0_ – T_A1_ respectively, for *N* = 1 with span Δt = 50 min. For *N* = 200 in a time interval [t_0_ + 95 min, t_0_ + 295 min] we found ρt_0_* = 5% and ρT_A0_* = 11% with the same Δt. Further research is necessary to guarantee applicability in routine casework, in particular with respect to more elaborate cooling models, estimation algorithms, and evaluation localization.

## Introduction

 Though temperature-based death time estimation (TTDE) is considered the most promising time of death estimation (TDE) method in forensic casework for short and medium times p.m., it is hampered by some serious and frequent drawbacks. Sudden drastic changes of the ambient temperature T_A_ during body cooling from a previous value T_A0_ to later observed T_A1_ (declines mostly) at some, often unknown, time t_0_ may lead to large errors in TTDE results (see [1, 2]) if not taken into account appropriately. Such changes in T_A_ may be caused by body transport as well as by opening doors or windows at the crime scene during the post mortem (p.m.) cooling phase before or after the body’s detection.

This problem is well known in forensic casework and recognized to be of importance. Several approaches to deal with changing ambient temperature have been developed, both for continuous changes [3] and for sudden changes [4, 5]. These studies, however, assume complete knowledge of the ambient temperature T_A_(t) as a function of time. In case of incomplete T_A_(t)-knowledge, there are attempts to estimate T_A_(t) from temperature measurements of T(t) on or in the cooling body [6–8]. Unfortunately, changes in time since death and in ambient temperature affect the later cooling in a similar way, rendering the estimation of time since death from body temperature measurements alone ill-conditioned.

Some heuristics for estimating at least T_A0_ have been proposed in the TTDE-literature [9, 10], such as closing the window and measuring T_A_ the next day, or T_A_-measurements in closed boxes Z or neighboring rooms of the crime scene. Such heuristics face two challenges: First, the time t_0_ at which the temperature drop occurred remains unknown, and second, the ambient temperature measured much later, or in a different room, need not correspond to the ambient temperature T_A0_ that affected corpse cooling prior to t_0_. In particular, closed boxes exposed to the temperature drop cool down as well, though much slower than the ambient temperature itself. Thus, one is confronted with the task of reconstructing T_A0_ from measurement results of T_Z_ in between T_A0_ and T_A1_.

Here, we aim at complete reconstruction of both T_A0_ and t_0_ from a sufficient number of temperature measurements in partially cooled, closed compartments. We describe the cooling of these compartments and their contents by Newtonian cooling, given mathematically as a simple exponential curve. This transforms the (T_A0_, t_0_)-reconstruction task into a well-defined parameter estimation problem. Our experiments in a climate chamber with closed compartments exposed to suddenly decreasing ambient temperature suggest that boxes with sufficient insulating air content cool down approximately in a Newtonian process. Estimating both T_A0_ and t_0_ requires at least two boxes with sufficiently different cooling curves and a minimum of four temperature measurements with two measurements taken in each of the boxes.

## Materials and methods

### Experiments

We perform two cooling experiments E1 and E2 in a climate chamber (Feutron 3706/06 RMA 3313). The chamber guarantees a constant ambient temperature T_A_(t) as a function of time t with a maximum deviation of +/−0.5 °C. In every experiment, two compartments (a cupboard and a cardboard box), called ‘boxes’ X and Y, respectively, in the following, are placed in the climate chamber before the start of the experiment. Box X contained air, while box Y contained books in experiment E1 and a heap of clothes in experiment E2. Both boxes were closed but not airtight, simulating common compartments like cupboards at a crime scene. All of our measurement equipment is from the series ALMEMO of the company AHLBORN. We place a temperature probe (FVAD 05-TOK300) in box X to measure the air temperature T_X_. Another sensor (FN0001K NTC-Sensor) is placed below the material in box Y to measure the temperature T_Y_ on the bottom of the box. Moreover, another temperature probe (FVAD 05-TOK300) is placed in the chamber next to the boxes to register the chambers ambient temperature T_A_. The temperatures T_A_, T_X_, T_Y_ are registered during the whole experiment every minute using data loggers (MA7120 and MA809).

Each experiment starts at t = 0 comprised two successive time intervals P0=[0, t_0_] and P1=[t_0_,t_end_]. During P0, the chamber is kept at a constant ambient temperature T_A0_. At time t_0_, the temperature is instantaneously downregulated from T_A0_ to a temperature T_A1_ and kept constant during P1.

**Fig. 1:**
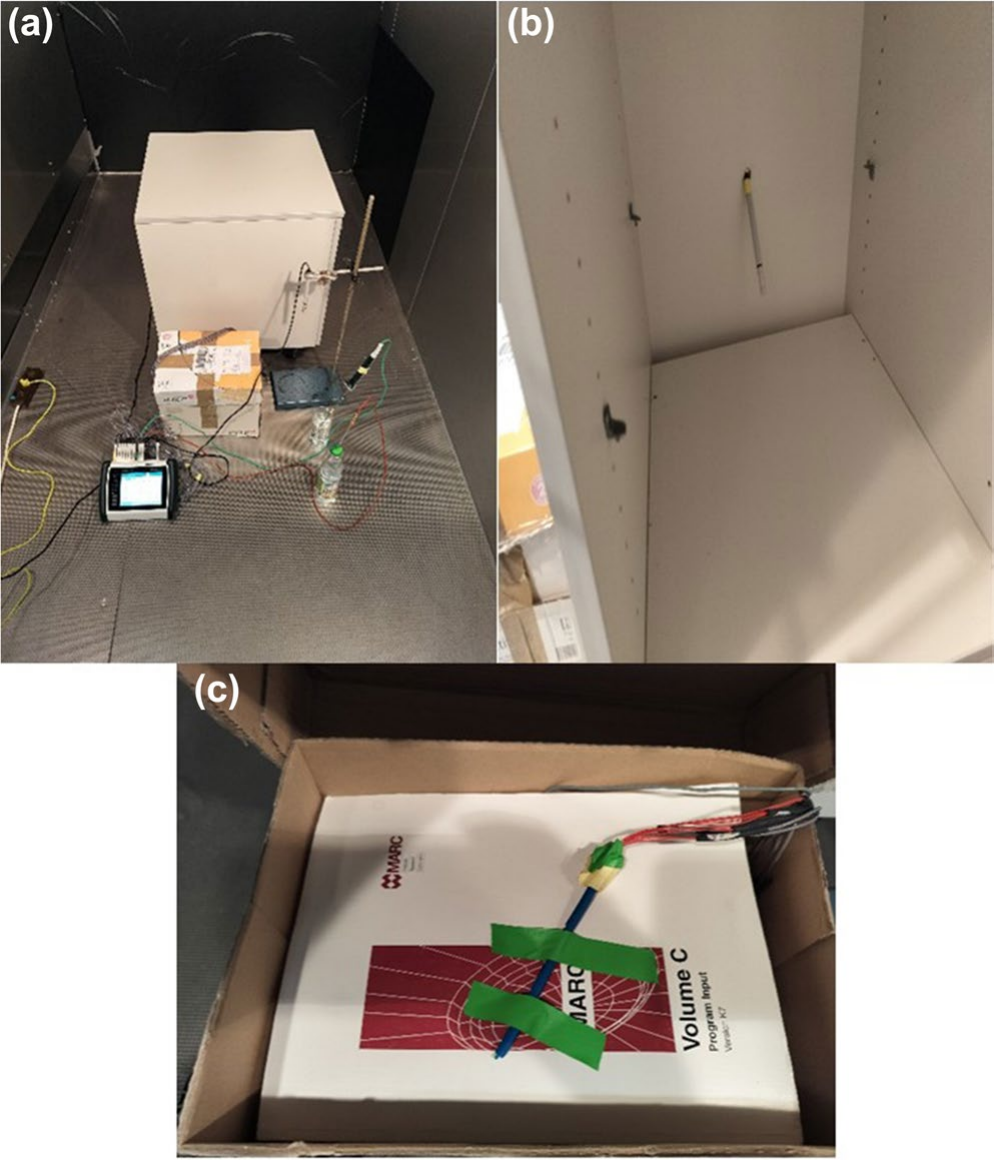
Experiment E1. **(a)** *top left*: Scenario in climate chamber with cupboard X (background) and cardboard box Y filled with books, ambient probe for T_A_, datalogger, and temperature probes in water bottles (not used for evaluation). **(b)** *top right*: Interior of cupboard X with probe for T_X_. **(c)** *bottom*: Interior of cardboard box Y with books and probe for T_Y_

 Figure 1 shows the experiment E1’s setup where Y is a cardboard box filled with books.

The parameters for the experiments E1 and E2 are as follows:

- E1: t_0_ = 305.00 min = 5 h 05 min; T_A0_ = 22.50 °C; T_A1_ = 14.00 °C.
- E2: t_0_ = 158.13 min = 2 h 38 min; T_A0_ = 22.16 °C; T_A1_ = 8.71 °C.

Though the E2 data did not exactly match our ideal scenario of two ambient temperature plateaus at time-constant temperatures T_A0_ and T_A1_ respectively, we kept the E2 data to test the robustness of our approach against deviations from this scenario, frequently met in application cases.

### Estimate of t_0_ and T_A0_ from four measurements of temperatures in boxes X and Y via Newtonian cooling

The following abbreviations and symbols are used:

- T_A0_ = Initial ambient temperature (unknown).
- T_A1_ = Constant ambient temperature during cooling (measured).
- T_X_(t) = Temperature inside closed box X (measured).
- T_Y_(t) = Temperature inside closed box Y (measured).
- T_Z,1_ = Temperature inside a closed box Z = X, Y at time t_1_ (measured).
- T_Z,2_ = Temperature inside a closed box Z = X, Y at time t_2_ (measured).
- t_0_ = Time of sudden temperature decrease: T_A0_ (unknown) -> T_A1_ (measured).
- t_1_ = Time of first two temperature measurements T_X,1_, T_Y_,_1_ during cooling.
- t_2_ = Time of second two temperature measurements T_X,2_, T_Y,2_ during cooling.
- Δt = Time difference between t_1_ and t_2_ called *quadruple span*.

**Fig. 2:**
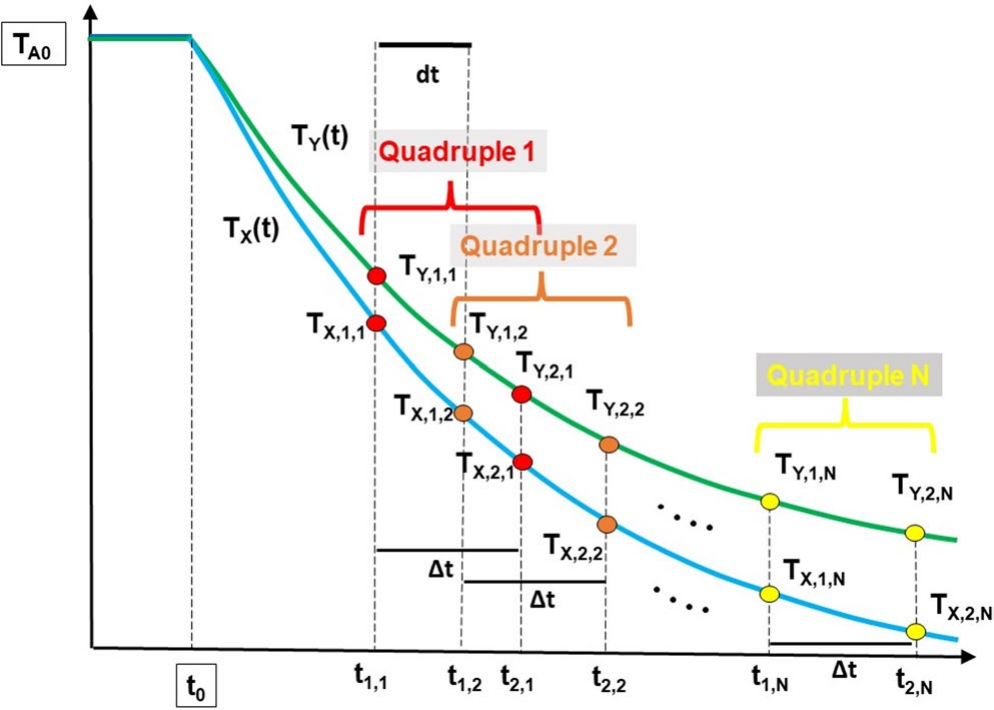
Initial external air temperature T_A0_, time t_0_ of decline T_A0_ to T_A1_, temperatures at time t in box X: T_X_(t) (blue), - in box Y: T_Y_(t) (green), measured temperatures in box Z = X, Y: T_Z, k,n_ k-th measurement at time t_k, n_ with k = 1, 2 and *n* = 1, …, N. Distance dt := t_1,*n*+1_ – t_1,n_ of neighboring quadruples for all *n*=1, …, N-1. Quadruple span Δt := t_1,n_ – t_2,n_ between time t_1,n_ of first temperature measurements T_X,1,n_, T_Y,1,n_ and time t_2,n_ of second temperature measurements T_X,2,n_ in n-th quadruple (t_1,n_, T_X,1,n_), (t_1,n_ T_Y,1,n_), (t_2,n_, T_X,2,n_), (t_n2,n_, T_Y,2,n_)

The measurements (t_1_, T_X_,_1_), (t_2_, T_X_,_2_), (t_1_, T_Y_,_1_), (t_2_, T_Y_,_2_) constitute one *quadruple* of temperature measurements. It is convenient though not necessary to choose two measurement times t_1_ and t_2_ only and to perform synchronous measurements T_X_,_1_, T_Y_,_1_ and T_X_,_2_, T_Y_,_2_, respectively, at each of the times t_1_ and t_2_ (see Fig. 2 with number N of quadruples. *N* = 1 for the single-quadruple case).

Newtonian cooling for T_Z_ = T_X_, T_Y_ at time t is given by

2.2.1 $$\frac{T_Z\left(t\right)-T_{A1}}{T_{A0}-T_{A1}}=e^{-a_Z\cdot(t-t_0)}$$

Taking the logarithm of (2.2.1) yields

2.2.2 $$\mathrm{ln}\left(T_Z\left(t\right)-T_{A1}\right)-\mathrm{ln}\left(T_{A0}-T_{A1}\right)=-a_Z\cdot(t-t_0)$$

Inserting T_Z_ = T_X,1_, T_X,2_ and t = t_1_, t_2_ into (2.2.2) and subtracting the two resulting equations: 

2.2.3 $$\mathrm{ln}\left(T_{X,1}-T_{A1}\right)-\mathrm{ln}\left(T_{A0}-T_{A1}\right)=-a_X\cdot(t_1-t_0)$$

2.2.4 $$\mathrm{ln}\left(T_{X,2}-T_{A1}\right)-\mathrm{ln}\left(T_{A0}-T_{A1}\right)=-a_X\cdot(t_2-t_0)$$

yields

2.2.5 $$\mathrm{ln}\left(T_{X,1}-T_{A1}\right)-\mathrm{ln}\left(T_{X,2}-T_{A1}\right)=-a_X\cdot(t_1-t_2)$$

and hence the factor a_X_:

2.2.6 $${a}_{X}=(\mathrm{ln}\left({T}_{X,1}-{T}_{A1}\right)-\mathrm{ln}\left({T}_{X,2}-{T}_{A1}\right))/({t}_{2}-{t}_{1})$$

Analogously, ${a}_{Y}$ is obtained from T_Z_ = T_Y,1_, T_Y,2_.

With known ${a}_{X},{a}_{Y}$, inserting ${T}_{Z}={T}_{X,1},{T}_{Y,1}$ into (2.2.2) results in:

2.2.7.X $$\mathrm{ln}\left({T}_{X,1}-{T}_{A1}\right)-\mathrm{ln}\left({T}_{A0}-{T}_{A1}\right)=-{a}_{X}\cdot({t}_{1}-{t}_{0})$$

2.2.7.Y $$\mathrm{ln}\left({T}_{Y,1}-{T}_{A1}\right)-\mathrm{ln}\left({T}_{A0}-{T}_{A1}\right)=-{a}_{Y}\cdot({t}_{1}-{t}_{0})$$

Subtracting (2.2.7.Y) from (2.2.7.X) yields:

2.2.8 $$\mathrm{ln}\left({T}_{X,1}-{T}_{A1}\right)-\mathrm{ln}\left({T}_{Y,1}-{T}_{A1}\right)=-{(a}_{X}-{a}_{Y})\cdot({t}_{1}-{t}_{0})$$

and thus an estimate for t_0_:

2.2.9 $${t}_{0}=\left[\mathrm{ln}\left({T}_{X,1}-{T}_{A1}\right)-\mathrm{ln}\left({T}_{Y,1}-{T}_{A1}\right)\right]/{(a}_{X}-{a}_{Y})+{t}_{1}$$

Finally, solving (2.2.1) for T_A0_ with ${T}_{Z}={T}_{X,1}$ yields

2.2.10 $${T}_{A0}=\left({T}_{X,1}-{T}_{A1}\right)\cdot{e}^{{a}_{Z}\cdot\left({t}_{1}-{t}_{0}\right)}+{T}_{A1}$$

Now, (2.2.6) can be used to estimate a_Z_, while (2.2.9) and (2.2.10) give single quadruple estimators t_0_^ and T_A0_^ for the unknown parameters t_0_ and T_A0_.

### Estimate from n quadruple measurements of temperature T in boxes X and Y via Newtonian cooling

Let T_A0_, T_A1_, t_0_ be as in Sect. 2.2, let further for all *n* = 1,…, N be t_1,n_ and t_2,n_ be the time points of temperature measurements T_X,1,n_, T_Y,1,n_ and T_X,2,n_, T_Y,2,n_ in the boxes X and Y, respectively, with a time distance Δt = t_2,n_ - t_1,n_ independent of n. The difference between the first measurement times t_1,n_ and t_1,(*n*+1)_ of two consecutive quadruples is dt = t_1,(*n*+1)_ – t_1,n_, which is chosen independently of n as well. Thus we have N = (t_1,N_ – t_1,1_)/dt + 1 (see Fig. 2).

The single estimators t_0,n_^ and T_A0,n_^ of the time t_0_ of temperature drop, and the ambient temperature T_A0_ before t_0_ are then computed as in Sect. 2.2:

2.3.1 $${t}_{0,n}^\wedge=\left[\mathrm{ln}\left({T}_{X,1,n}-{T}_{A1}\right)-\mathrm{ln}\left({T}_{Y,2,n}-{T}_{A1}\right)\right]/{(a}_{X,n}^\wedge-{a}_{Y,n}^\wedge)+{t}_{1,n}$$

2.3.2 $${T}_{A0,n}^\wedge=\left({T}_{Z,1,n}-{T}_{A1}\right)\cdot{e}^{{a}_{Z,n}^\wedge\cdot\left({t}_{1,n}-{t}_{0}\right)}+{T}_{A1}$$

The Weighted Mean Estimators (WME) t_0_* and T_A0_* are obtained by computing the weighted mean of all of the respective single estimators t_0,n_^ and T_A0,n_^ from the N equations in (2.3.1) and in (2.3.2), respectively, with weight factors W_1_, …, W_N_ for the N equations in (2.3.1) and in (2.3.2). Weighting of the mean computation with the factors W_t,__n_ and W_T,n_ gives the WMEs t_0_* and T_A0_* respectively:

2.3.3 $$t_0^\ast\mathit:=\sum\nolimits_{n=1,...,N}\;W_{t,n}\cdot\;t_{0,\;n}^\wedge$$

2.3.4 $$T_{A0}^\ast\;:=\sum\nolimits_{n=1,...,N}\;W_{T,n}\;\cdot\;T_{A0,\;n}^\wedge$$

Choosing the weights W_t,__n_ and W_T,n_ inversely proportional to the standard deviations St_0,n_^ and ST_A0,n_^ of the single estimators t_0,n_^ and T_A0,n_^ in the equations (2.3.1) and (2.3.2), respectively, is the canonical approach, and yields

2.3.5 $$W_{t,\;n}\sim1/St_{0,n}^\wedge$$

2.3.6 $$W_{T,n}\sim1/ST_{A0,\;n}^\wedge$$

with

2.3.7 $$\forall n=1,...,N:0\leq W_{t,\;n},\;W_{T,n}\leq1$$

and

2.3.8 $$1=\sum\nolimits_{n=1,\dots,N}{W}_{t,n}=\sum\nolimits_{n=1,\dots,N}{W}_{T,n}$$

The time difference between the measurement times t_1,n_ and t_1,(*n*+1)_ of two consecutive quadruples with indices n and *n* + 1 is named dt and *is kept constant in all of our experiments and computations*:

2.3.9 $$dt\;:=t_{1,\left(n+1\right)}-t_{1,n}$$

### Estimate weights and errors of the method

The usual Gaussian error propagation approach – estimating standard deviations via linear approximation of random variables - yields the standard deviation estimators St_0_^ and ST_A0_^ for the single quadruple approach as well as St_0_* and ST_A0_* for the WMEs with multiple quadruple measurements. The standard deviation estimations are used for error quantification as well as for weight computation in the WME. We assume the errors of the input random variables T_A1_, T_X,1_, T_X,2_, T_Y,1,_ T_Y,2_, t_1_, t_2_ to be independently and identically normally distributed with standard deviation ST = 0.1 °C for temperature variables T and St = 1 min for time variables t. Denoting the true values by t_0_^+^ and T_A0_^+^, and the estimated values by t_0_^#^ (= t_0_^ or t_0_*) and T_A0_^#^ (= T_A0_^ or T_A0_*), we can write the errors δt_0_^#^ and δT_A0_^#^ as

2.4.1 $$\delta t_0^\#\;:=t_0^\#-t_0^+$$

2.4.2 $$\delta T_{A0}^\#:=T_{A0}^\#-T_{A0}^+$$

With fixed values for N and the time difference Δt, the relative errors ρt_0_^#^, ρT_A0_^#^ are computed as

2.4.3 $$\rho t_0^\#\left(t\right)\;:=\delta t_0^\#/(t-t_0^+)$$

2.4.4 $$\rho{{T}_{A0}}^{\#}\left({t}\right):=\delta{{T}_{A0}}^{\#}/({T}_{A0}^{+}-{T}_{A1})$$

In (2.4.3) and (2.4.4) as well as in (2.4.5), the variable t has to be substituted by t_1_ in case N = 1 and by t_1,1_ in case N > 1. The relative standard deviations Rt_0_^#^, RT_A0_^#^ are computed from the standard deviations St_0_^#^, ST_A0_^#^ as

2.4.5 $${R{t}_{0}}^{\#}:=S{{t}_{0}}^{\#}/({t}-{t}_{0}^{+})$$

2.4.6 $${R{T}_{A0}}^{\#}:=S{{T}_{A0}}^{\#}/({T}_{A0}^{+}-{T}_{A1})$$

The errors δt_0_^#^, δT_A0_^#^, the standard deviations St_0_^#^, ST_A0_^#^, the relative errors ρt_0_^#^, ρT_A0_^#^, and the relative standard deviations Rt_0_^#^, RT_A0_^#^ all depend on the *design parameters* N, Δt, dt, t_1_ or t_1,1_ respectively. Mostly, we will omit the dependency on the design parameters in the symbols. If we want to emphasize the dependency on one of the design parameters, we denote it as argument of the respective symbol explicitly, as, e.g., in RT_A0_*(t_1,N_).

## Results

### Evaluation with one measurement quadruple

**Fig. 3:**
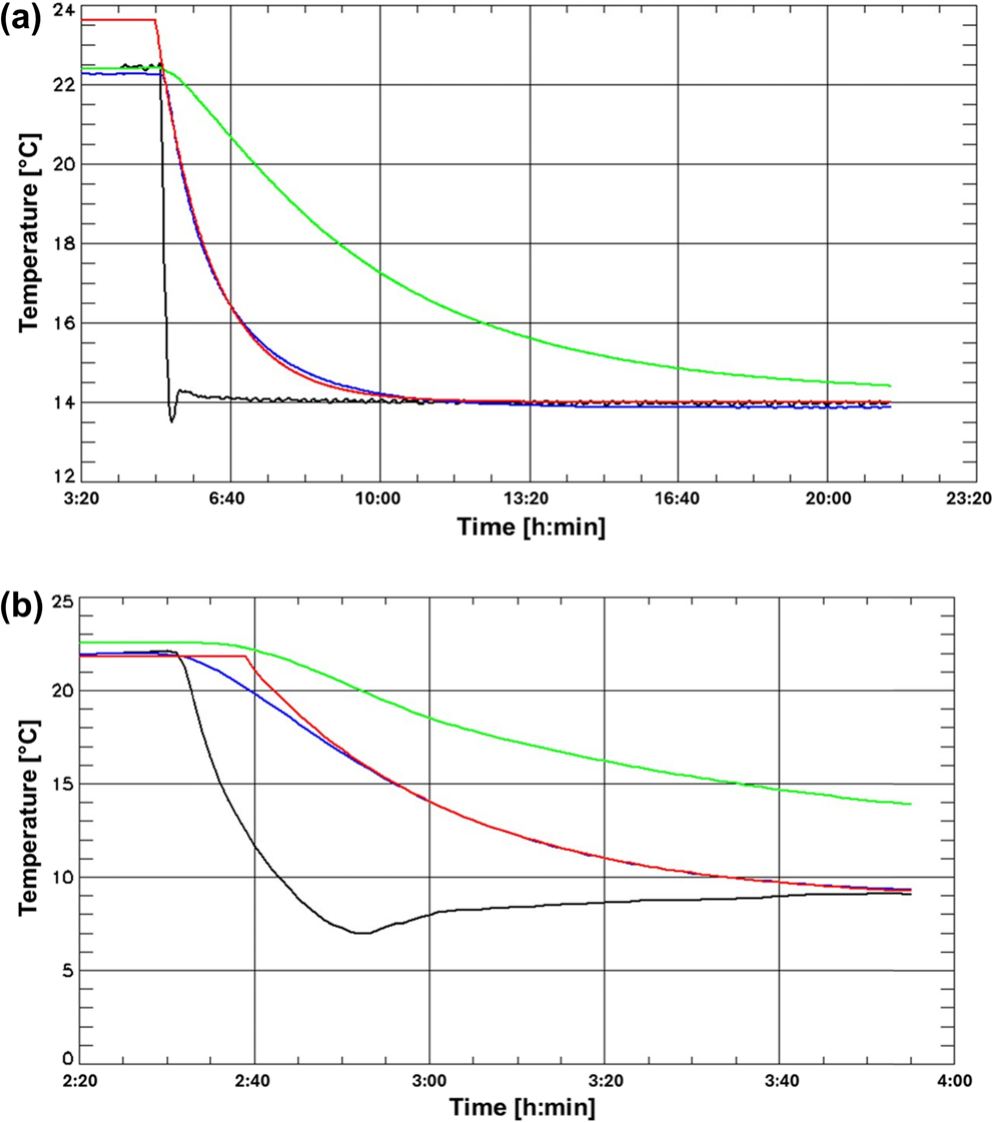
**(a)** *top*: Experiment E1, **(b)** *bottom*: Experiment E2. Measured curves: ambient temperature T_A_(t) (black), air temperature T_X_(t) (blue) in box X, temperature T_Y_(t) (green) on the bottom of box Y below materials (E1: books, E2: pile of clothes). The reconstructed cooling curve of T_X_^ (red) demonstrates the estimated values T_A0_^ and t_0_^ for single measurement quadruple estimation. The measurement times were t_1_ = 6 h 40 min, t_2_ = 10 h 50 min (Δt = 4 h 10 min) for E1 and t_1_ = 3 h and t_2_ = 3 h 10 min (Δt = 10 min) for E2 respectively

Figure 3 shows the measured temperature curves T_A_(t) (black), T_X_(t) (blue), T_Y_(t) (green) for the experiments E1 and E2 respectively, as well as the curve T_X_^(t) (red) computed via (2.2.1) using the parameter estimation results t_0_^ and T_A0_^, respectively. For E1, the times t_1_, t_2_ for the respective two measurements are t_1_ = 6 h 40 min, t_2_ = 1_1_0 h 50 min, whereas for E2 we choose t_1_ = 3 h and t_2_ = 3 h 10 min.

The results of all one-quadruple estimations are represented in Table 1 for E1 and in Table 2 for E2.

**Table 1:** Results of estimations in E1 for only one measurement quadruple. Quadruple span Δt, first measurement time t_1_ and last measurement time t_2_ of the first quadruple; estimation values t_0_^ for time limit t_0_ and T_A0_^ for temperature T_A0_ of first interval P0; relative errors ρt_0_^ and ρT_A0_^ and relative standard deviations Rt_0_^ and RT_A0_^ of the estimators

Δt [h: min]	t_1_ [h: min]	t_2_ [h: min]	t_0_^ [h: min]	T_A0_^ [°C]	ρt_0_^ [1]	ρT_A0_^ [1]	Rt_0_^ [1]	RT_A0_^ [1]
0:10	5:50	6:00	5:01.97	22.88	−0.14	0.05	0.44	0.18
0:20	5:50	6:10	5:00.57	22.94	−0.18	0.05	0.25	0.10
0:50	5:50	6:40	4:58.22	23.07	−0.15	0.09	0.15	0.05
1:40	5:50	7:30	4:53.88	23.30	−0.25	0.09	0.12	0.03
2:30	5:50	8:20	4:50.88	23.46	−0.31	0.11	0.14	0.03
3:20	5:50	9:10	4:50.46	23.52	−0.32	0.12	0.19	0.03
4:10	5:50	10:00	4:51.99	23.50	−0.29	0.12	0.29	0.04
5:00	5:50	10:50	4:58.68	23.30	−0.14	0.09	0.46	0.06
0:10	6:40	6:50	4:43.63	23.94	−0.26	0.17	0.70	0.42
0:20	6:40	7:00	4:44.15	23.89	−0.26	0.16	0.39	0.22
0:50	6:40	7:30	4:39.60	24.12	−0.27	0.19	0.21	0.11
1:40	6:40	8:20	4:34.94	24.38	−0.32	0.22	0.19	0.07
2:30	6:40	9:10	4:36.82	24.38	−0.30	0.22	0.24	0.08
3:20	6:40	10:00	4:42.44	24.21	−0.24	0.20	0.35	0.10
4:10	6:40	10:50	4:59.30	23.63	−0.06	0.13	0.54	0.15
0:10	8:20	8:30	5:00.23	23.93	−0.04	0.17	0.23	0.16
0:20	8:20	8:40	4:42.45	24.50	−0.13	0.24	0.13	0.87
0:50	8:20	9:10	4:44.83	24.39	−0.10	0.22	0.70	0.45
1:40	8:20	10:00	5:00.41	23.82	−0.02	0.16	0.66	0.40
2:30	8:20	10:50	5:39.60	22.50	0.18	0.00	0.86	0.50

**Table 2:** Results of estimations in E2 for only one measurement quadruple: Quadruple span Δt, first measurement time t_1_ and last measurement time t_2_ of the first quadruple; estimator values t_0_^ for time limit t_0_ and T_A0_^ for temperature T_A0_ of first interval P0, as well as relative errors ρt_0_^ and ρT_A0_^ and relative standard deviations Rt_0_^ and RT_A0_^ of the estimators

Δt [h: min]	t_1_ [h: min]	t_2_ [h: min]	t_0_^ [h: min]	T_A0_^ [°C]	ρt_0_^ [1]	ρT_A0_^ [1]	Rt_0_^ [1]	RT_A0_^ [1]
0:10	3:00	3:10	2:38.13	22.16	0.00	0.00	0.24	0.20
0:20	3:00	3:20	2:38.61	21.83	0.02	−0.02	0.16	0.11
0:30	3:00	3:30	2:39.29	21.55	0.05	−0.05	0.14	0.08
0:40	3:00	3:40	2:39.07	21.47	0.04	−0.05	0.14	0.06
0:50	3:00	3:50	2:38.64	21.41	0.02	−0.06	0.16	0.06
0:10	3:20	3:30	2:42.31	20.39	0.10	−0.13	0.36	0.43
0:20	3:20	3:40	2:40.34	20.52	0.05	−0.12	0.25	0.24
0:30	3:20	3:50	2:38.70	20.51	0.01	−0.12	0.24	0.19
0:10	3:40	3:50	2:32.25	20.50	−0.10	−0.12	0.73	0.86

### Error estimation for one quadruple measurement

**Fig. 4:**
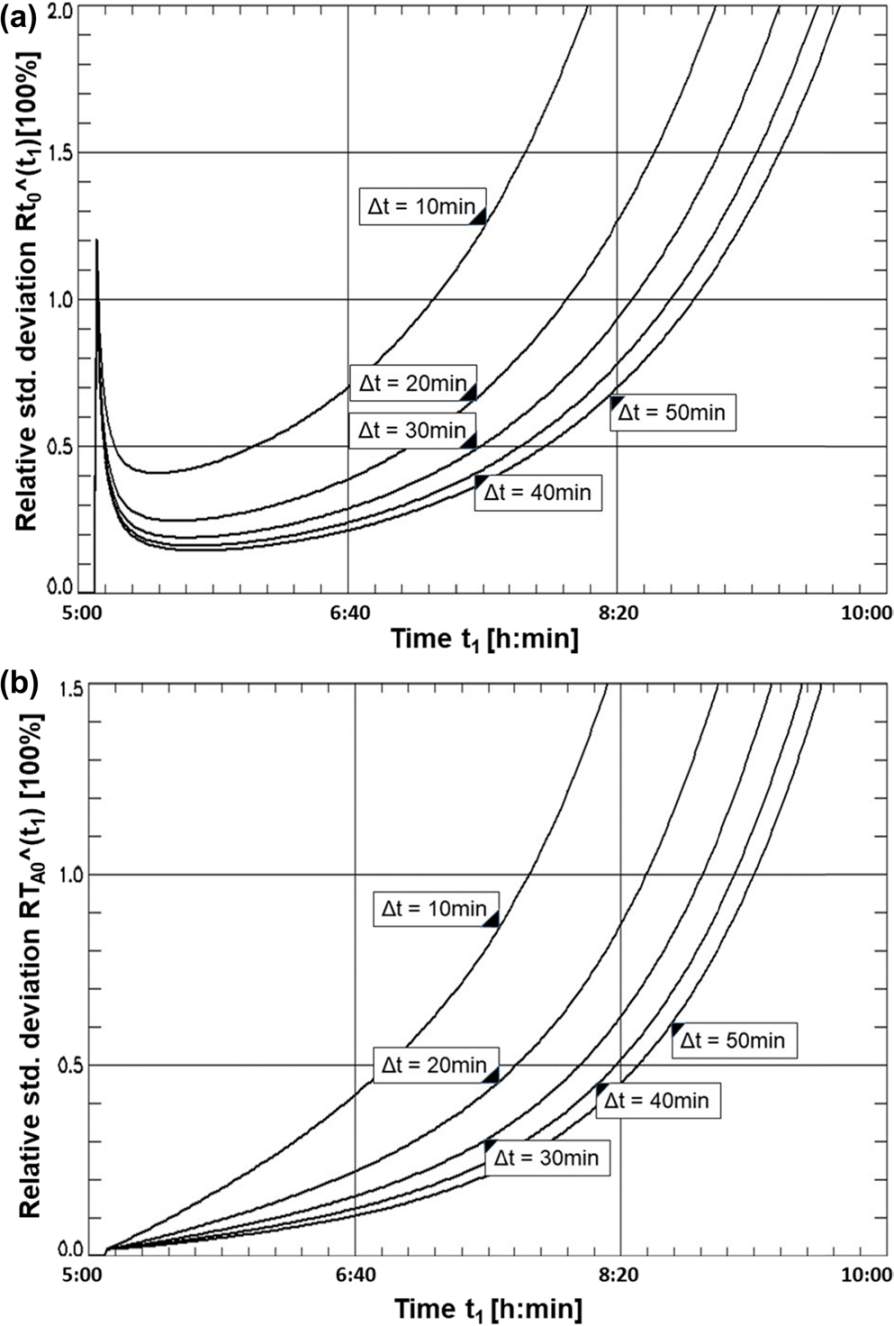
For experiment E1: Relative standard deviations Rt_0_^(t_1_) (in **(a)** *top*) and RT_A0_^(t_1_) (in **(b)** *bottom*) as functions of the first measurement position t_1_ for different quadruple spans Δt = t_2_ – t_1_ as given in the labels

**Fig. 5:**
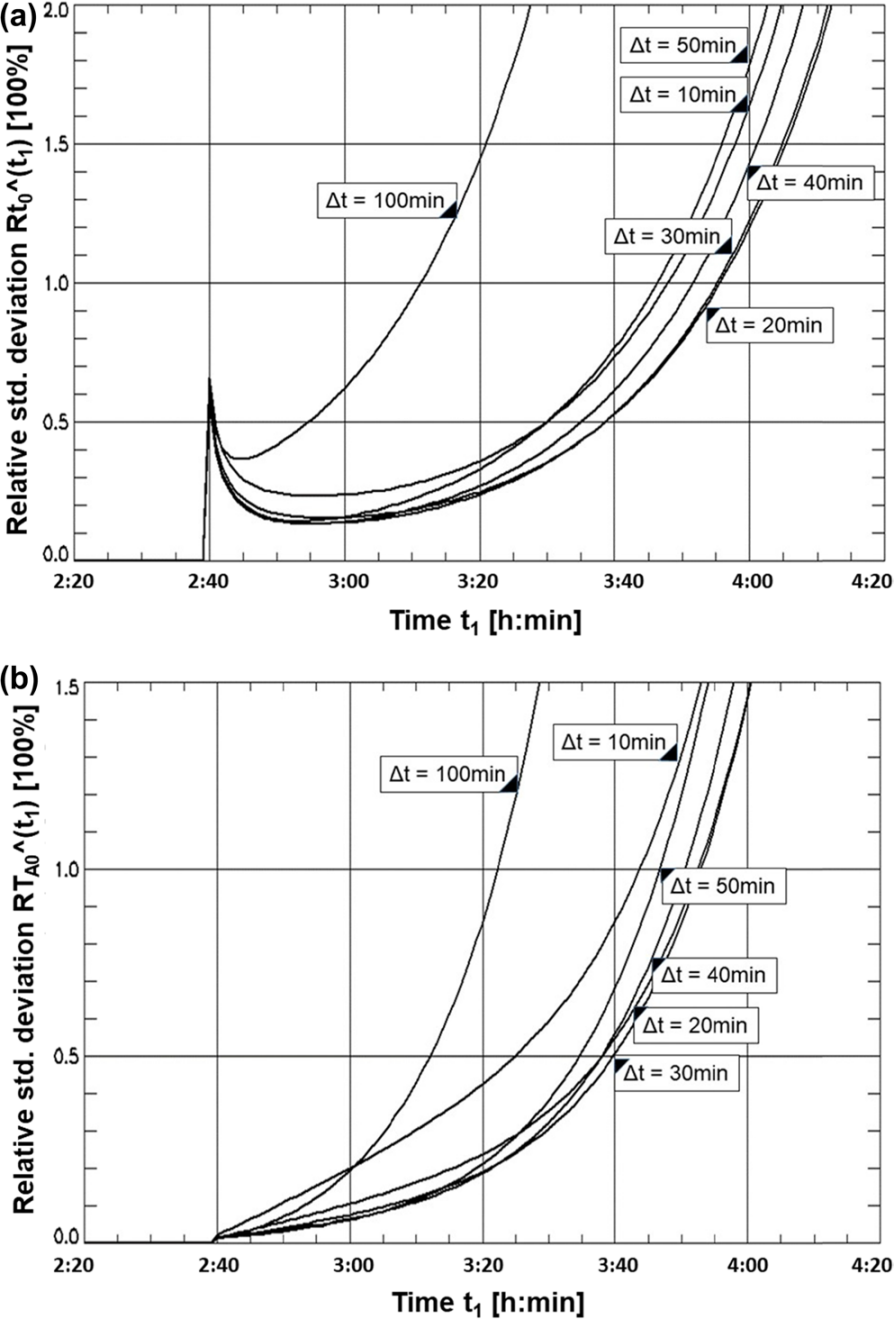
For experiment E2: Relative standard deviations Rt0^(t1) (in (a) top) and RTA0^(t1) (in (b) bottom) as a function of the first measurement position t1,1 for different quadruple spans Δt = t2 – t1 as given in the labels

In Fig. 4 and in Fig. 5 the relative standard deviations Rt_0_^ and RT_A0_^ are shown as functions of the first measurement time t_1_. The diagrams present the curves for fixed quadruple spans Δt = 10 min, 20 min, 30 min, 40 min, 50 min in experiment E1 (see Fig. 4) and for Δt = 10 min, 20 min, 30 min, 40 min, 1 h 40 min in experiment E2 (see Fig. 5). The coefficients a_X_ and a_Y_ are set to:

- a_X_ = 0.01288 min^−1^, a_Y_ = 0.00298 min^−1^ for E1 obtained in  subsection "Evaluation with one measurement quadruple" with t_1_ = 5 h 50 min, t_2_ = 6 h 40 min.

- a_X_ = 0.04204 min^−1^, a_Y_ = 0.01428 min^−1^ for E2 obtained in subsection "Evaluation with one measurement quadruple" with t_1_ = 3 h, t_2_ = 3 h 10 min.

### Evaluation with several measurement quadruples

The evaluation with several measurement quadruples is carried out with varying times of first quadruple measurement t_1,1_ and varying last quadruple measurement time t_1,N_. As the time interval between quadruples, dt = 1 min, remains constant, the resulting number N of quadruples per evaluation varies accordingly.

**Fig. 6:**
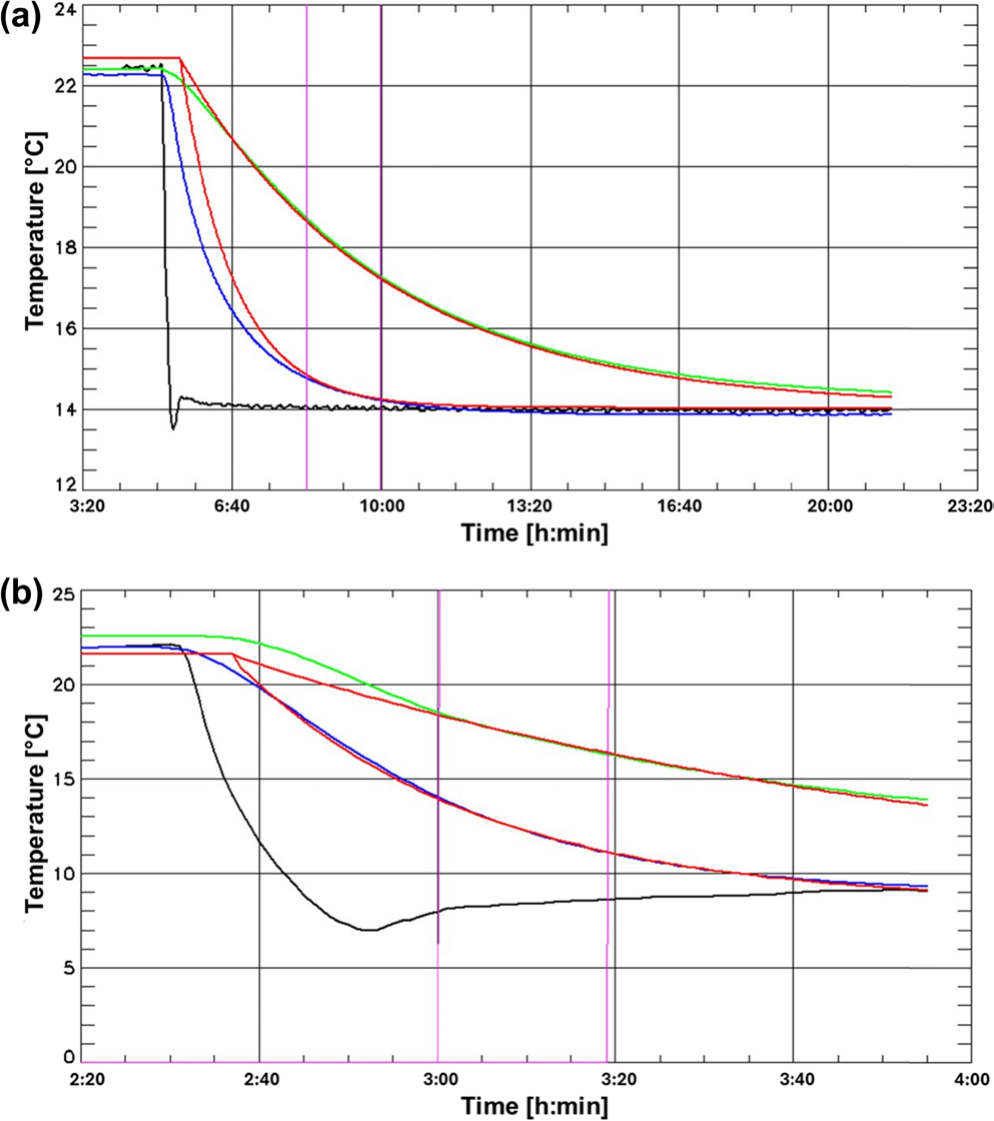
In (a) top: Experiment E1, in (b) bottom: Experiment E2. Measured curves: ambient temperature TA(t) (black), air temperature TX(t) (blue) in box X, temperature TY(t) (green) on the bottom of box Y below materials (E1: books, E2: pile of clothes). The reconstructed cooling curves of TX* and TY* (red) demonstrate the estimated values TA0* and t0* for multiple measurement quadruple estimation. The points in time t1,1 and t1,N of measurement begin for the first and for the last quadruple are marked by thin rectangular lines (pink). The distance between adjacent quadruples is dt = 1min and the number N = (t1,N – t1,1) / dt + 1 of quadruples varies accordingly

Figure 6 presents an example of estimation results for approaches with several measurement quadruples: The two temperature-time diagrams present the measured curves T_A_(t) (black), T_X_(t) (blue), T_Y_(t) (green) for the experiments E1 and E2 respectively. The curves T_X_*(t) (red) and T_Y_*(t) (red) are reconstructed using the estimator values a_X_* and a_Y_*, which are interim results of computing the final estimation results t_0_* and T_A0_*. For E1 the first quadruple’s first measurement time is t_1,1_ = 8 h 20 min (pink mark left) while the last quadruple’s first measurement time is t_1,N_ = 9 h 59 min (pink mark right), which adds up to *N* = 100 quadruples. For E2, the number of measurement quadruples is *N* = 20 with the first measurement time t_1,1_ = 3 h (pink mark left) of the first quadruple and the last quadruples first measurement at t_1,N_ = 3 h 19 min (pink mark right). The quadruple span Δt = t_2,n_ – t_1,n_ is Δt = 20 min for all quadruples, while the time distance dt = t_1,(*n*+1)_ – t_1,n_ between the first measurements of two successive quadruples is dt = 1 min for all evaluations.

The results of all *N* > 1 quadruple-estimations are represented in Table 3 for E1 and in Table 4 for E2.

**Table 3:** Results of estimations in E1 for *N* > 1 measurement quadruples. Quadruple span Δt, first measurement time t_1,1_ of first quadruple and first measurement time t_1,N_ of the last quadruple; estimation values t_0_* for time limit t_0_ and T_A0_* for temperature T_A0_. Relative errors ρt_0_* and ρT_A0_* and relative standard deviations Rt_0_* and RT_A0_* of the estimators. The distance between neighbor-quadrupels is dt = 1 min and the number N = (t_1,N_ – t_1,1_)/dt + 1 of quadruples varies accordingly

Δt [h: min]	t_1,1_ [h: min]	t_1,*N*_ [h: min]	t_0_* [h: min]	T_A0_* [°C]	ρt_0_* [1]	ρT_A0_* [1]	Rt_0_* [1]	RT_A0_* [1]
0:10	5:50	6:39	4:58.11	23.13	−0.15	0.07	0.06	0.05
0:10	5:50	8:19	4:54.17	23.46	−0.24	0.11	0.04	0.05
0:10	5:50	9:59	4:57.02	23.33	−0.18	0.10	0.03	0.06
0:10	6:40	8:19	4:43.37	24.12	−0.23	0.19	0.10	0.12
0:10	6:40	9:59	4:52.61	23.72	−0.13	0.14	0.08	0.13
0:10	7:30	8:19	4:40.08	24.42	−0.17	0.23	0.26	0.27
0:10	7:30	9:59	5:01.68	23.54	−0.02	0.12	0.16	0.22
0:10	8:20	9:59	5:24.15	22.87	0.10	0.04	0.28	0.35
0:50	5:50	6:39	4:54.32	23.37	−0.24	0.10	0.02	0.01
0:50	5:50	8:19	4:51.32	23.65	−0.30	0.14	0.01	0.01
0:50	5:50	9:59	4:57.60	23.31	−0.16	0.09	0.01	0.02
0:50	6:40	8:19	4:43.65	24.19	−0.22	0.20	0.03	0.03
0:50	6:40	9:59	5:00.64	23.40	−0.05	0.11	0.02	0.03
0:50	7:30	8:19	4:45.77	24.28	−0.13	0.21	0.07	0.07
0:50	7:30	9:59	5:19.94	22.85	0.10	0.04	0.04	0.05
0:50	8:20	9:59	5:53.09	21.91	0.25	−0.07	0.07	0.08

**Table 4:** Results of estimations in E2 for *N* > 1 measurement quadruples. Quadruple span Δt, first measurement time t_1,1_ of quadruple 1 and first measurement time t_1,N_ of the last quadruple; estimation values t_0_* for time limit t_0_ and T_A0_* for temperature T_A0_. Relative errors ρt_0_* and ρT_A0_* and relative standard deviations Rt_0_* and RT_A0_* of the estimators. The distance between neighbor-quadruples is dt = 1 min and the number N = (t_1,N_ – t_1,1_)/dt + 1 of quadruples varies accordingly

Δt [h: min]	t_1,1_ [h: min]	t_1,*N*_ [h: min]	t_0_* [h: min]	T_A0_* [°C]	ρt_0_* [1]	ρT_A0_* [1]	Rt_0_* [1]	RT_A0_* [1]
0:10	3:00	3:09	2:36.98	21.93	−0.05	−0.02	0.07	0.07
0:10	3:00	3:19	2:37.18	21.71	−0.04	−0.03	0.05	0.06
0:10	3:00	3:29	2:37.65	21.44	−0.02	−0.05	0.04	0.05
0:10	3:00	3:39	2:37.86	21.29	−0.01	−0.06	0.03	0.05
0:10	3:20	3:29	2:41.41	20.32	0.08	−0.14	0.10	0.11
0:10	3:20	3:39	2:42.05	20.07	0.09	−0.16	0.08	0.09

## Discussion

Errors in ambient temperature T_A_ are of extreme importance in TTDE (see e.g. [1, 2]). There have been several attempts to deal with or even to overcome this problem. In practical TTDE casework, there are frequent scenarios, where a constant indoor ambient temperature is significantly reduced by scene manipulations (see [11]). The latter sudden temperature drops typically occur during indoor homicide investigations when crime scene investigators, unaware of the thermodynamical consequences, open windows for ventilation before the ambient temperature is measured.

One approach [8] to deal with said situations shows under the assumption of the Marshall & Hoare model with parameter determination of Henßge (MHH) that the information about a sudden T_A_-decline during body cooling is contained in the cooling curve itself and can in principle be extracted numerically. Including additional external information is, however, the best way to improve the reconstruction accuracy. We use temperature measurements in closed compartments exposed to a sudden ambient temperature drop T_A0_ =>T_A1_ at a time t_0_ in a climate chamber to test a method of reconstructing the time t_0_ of change and the previous external ambient temperature T_A0_ from a posteriori temperature measurements in the boxes. Our ultimate goal is to apply the method at crime scenes in cases where the information about amount and time of a recent p.m. ambient temperature drop is unknown. The estimated values of t_0_ and T_A0_ can serve as input for TTDE using, e.g., a Finite Element Model (FEM) as in [12, 13].

Note that using a single box is not sufficient for estimating both t_0_ and T_A0_. Graphically, we see that the cooling curve of a single box can uniquely be identified from two sequential measurements, but the curve itself is compatible with a continuum of possible times t_0_ and corresponding temperatures T_A0_. Two different cooling curves are required for determining their intersection point (t_0_, T_A0_), as shown in Fig. 2.

In the following, it is important to note that the standard deviations St_0_^#^ and ST_A0_^#^ required for Rt_0_^#^ and RT_A0_^#^ are computed using Gaussian error propagation, assuming compatibility of the Newtonian cooling model. Therefore, the relative errors ρt_0_^#^ and ρT_A0_^#^ are more suitable indicators of model quality than the relative standard deviations Rt_0_^#^ and RT_A0_^#^ because they are including the estimators’ bias as well.

Our following conclusions (in italics) about interactions of the design-parameters with accuracy and exactness are induced by the computation results. The reference to Tables is abbreviated, with “(Tn, Lk)” meaning “Tab. N, line k”.

*The usage of N > 1 quadruple measurments seems to improve reconstruction quality in terms of decreasing values of* ρ*t*_0_*, ρ*T*_A0_*, R*t*_0_*, R*T*_A0_* in comparison to the single quadruple approach’s (N= 1) values ρ*t*_*0*_*^*, ρ*T*_*A0*_*^*, R*t*_*0*_*^*, R*T*_*A0*_*^*:

In E1-reconstruction, where t_0_ = 5:05 the following examples give evidence.

T1 L11 $$\begin{array}{c}N=1,\;t_1=6:40\;min=\;t_0+1h\;45\;min,\triangle t=50min,\;\rho t_{0^\wedge}=\;-27\%,\;\rho T_{A0^\wedge}=19\%,\;Rt_{0^\wedge}=21\%,\;RT_{A0^\wedge}=11\%\end{array}$$

T3 L13 $$\begin{array}{c}N=200,\;t_{1,1}=6:40min,\;t_{1,N}=9:59,\;\triangle t=50min,\;\rho t_0^\ast=-5\%,\;\rho T_{A0^\ast}=11\%,\;Rt_{0^\ast}=2\%,\;RT_{A0^\ast}=3\%\end{array}$$

This could imply rising N to reduce relative errors and relative standard deviations.

Comparing:

T1 L9 $$\begin{array}{c}N=1,\;t_1=6:40min=t_0+\;1h\;45min,\;\triangle t=10min,\rho t_{0^\wedge}=-26\%,\;\rho T_{A0^\wedge}=17\%,\;Rt_{0^\wedge}=70\%,\;RT_{A0^\wedge}=42\%\\\end{array}$$

T3 L4 $$\begin{array}{c}N=100,\;t_{1,1}=6:40,\;t_{1,\;N}=8:19,\triangle t=10min,\;\rho t_{0^\wedge}=-23\%,\;\rho T_{A0^\wedge}\;=19\%,\;Rt_{0^\ast}=10\%,\;RT_{A0^\ast}=12\%\end{array}$$

shows, that at least the relative standard deviations are reduced by rising N for small Δt-values.

The next comparison:

T1 L18 $$\begin{array}{c}N=1,\;t_1=8:20\;=t_0+3h\;15\;min,\;\triangle t=50min,\;\rho t_{0^\wedge}=-10\%,\;\rho T_{A0^\wedge}=22\%,\;Rt_{0^\wedge}=70\%,\;RT_{A0^\wedge}=45\%\end{array}$$

T3 L16 $$\begin{array}{c}N=100,\;t_{1,1}=8:20,\;t_{1,\;N}=9:59,\triangle t=50min,\;\rho t_{0^\wedge}=25\%,\;\rho T_{A0^\wedge}\;=-7\%,\;Rt_{0^\ast}=7\%,\;RT_{A0^\ast}=8\%\end{array}$$

presents an example, where rising N reduced relative T_A0_-error and relative standard deviations for larger t_1_ than in the examples before.

*Increasing values of t*_*1*_ *or t*_*1,1*_, *which means later measurement start times*,* generally leads to larger values of Rt*_*0*_^*#*^ *and RT*_*A0*_^*#*^:

For *N* = 1 this can be seen in the diagrams of Figs. 4 and 5: All RT_A0_^ curves (Fig. 4b for E1 and Fig. 5b for E2) and for values t_1_ > 5 h 40 min in (Fig. 4a for E1) as well as for t_1_ > 2 h 50 min in (Fig. 5 for E2) all of the Rt_0_^ curves are monotonically increasing with rising measurement begin t_1_.

For *N* > 1 we see in Table 3 for E1 monotonically increasing values of Rt_0_*(t_1,1_) and RT_A0_*(t_1,1_) for increasing t_1,1_ e.g.:

T3 L4 $$\begin{array}{c}N=100,\;\triangle t=10min,\;t_{1,1}=6h\;40min,\;t_{1,\;N}=8h\;19min,Rt_{0^\ast}\left(t_{1,1}\right)=10\%,\;RT_{A0^\ast}\left(t_{1,1}\right)=12\%\end{array}$$

T3 L8 $$\begin{array}{c}N=100,\;\triangle t=10min,\;t_{1,1}=\;8h\;20min,\;t_{1,N}=9h\;59min,Rt_{0^\ast}\left(t_{1,1}\right)=28\%,\;RT_{A0^\ast}\left(t_{1,1}\right)=35\%\end{array}$$

Table 4 shows the same effect for E2, e.g.:

T3 L1 $$\begin{array}{c}N=10,\;\triangle t=10min,\;t_{1,1}=3h\;00min,\;t_{1,N}=3h\;09min,\;Rt_{0^\ast}\left(t_{1,1}\right)=7\%,\;RT_{A0^\ast}\left(t_{1,1}\right)=7\%\end{array}$$

T3 L5 $$\begin{array}{c}N=10,\;\triangle t=10min,\;t_{1,1}=3h\;20min,\;t_{1,N}=\;3h\;29\;min,\;Rt_{0^\ast}\left(t_{1,1}\right)=10\%,\;RT_{A0^\ast}\left(t_{1,1}\right)=11\%\end{array}$$

We notice that in E1 larger quadruple spans Δt generally lead to smaller Rt_0_^#^ and RT_A0_^#^. We conjecture that this dependency is disturbed in experiment E2 (see Fig. 6) due to the experimental deviation of T_A_(t) from our model assumption of two adjacent plateaus with constant temperatures T_A0_ and T_A1_ respectively and an instantaneous decrease at t_0_ between them.

For *N* = 1 this is implied by Fig. 4a for Rt_0_^#^ and by Fig. 4b for RT_A0_^#^ as the curves Rt_0_^#^(t_1_) and RT_A0_^#^(t_1_) are sorted with respect to Δt: Larger Δt-values are associated with lower Rt_0_^#^(t_1_) and RT_A0_^#^(t_1_) for all t_1_.

For multiple quadruple measurements *N* > 1 the numbers in Table 3 show the same trend, e.g.:

T3 L1 $$\begin{array}{c}N=50,\;\triangle t\:=10\;min,\;t_{1,1}=5h\;50min,t_{1,N}=6h\;39min,\;Rt_{0\ast}=\;6\%,\;RT_{A0\ast}=\;5\%\end{array}$$

T3 L9 $$\begin{array}{c}N=50,\;\triangle t\:=50min,\;t_{1,1}=5h\;50min,\;t_{1,N}\;=\;6h\;39min,\;R_{t0\ast}=\;2\%,\;RT_{A0\ast}=1\%\end{array}$$

The estimators t_0_^ and T_A0_^, respectively, seem to have a negative respective positive bias due to Newtonian model misfit to the data.

Looking at the algebraic signs of the *N* = 1 quadruple estimators’ t_0_^ and T_A0_^ relative errors ρt_0_^ and ρT_A0_^ in Table 1 for E1, we see nearly constant negatives in case of t_0_ and a nearly constant positive relative error for T_A0_. This is reversed in Table 2 for E2.

The discrepancy between the relative errors ρt_0_* and ρT_A0_* compared to the relative standard deviations Rt_0_* and RT_A0_*, respectively, in Table 3 and Table 4 appears to stem from our Newtonian model assumption as well.

Assuming the error distributions to be Gaussians and the Newtonian model to be without bias compared to our data, we would expect around q = 0.68 = 68% of the relative errors ρt_0_*(t_1_) and ρT_A0_*(t_1_) to fall into the intervals It_0_*(t_1_) := [-Rt_0_*(t_1_), Rt_0_*(t_1_)] and IT_A0_*(t_1_) := [-RT_A0_*(t_1_), RT_A0_*(t_1_)] respectively. In Table 3 (for E1) the condition “ρt_0_*(t_1_) in It_0_*(t1)” is violated in m = 13 out of M = 16 cases (81.2%), and m = 11 of M = 16 cases (68.8%) fail “ρT_A0_*(t_1_) in IT_A0_*(t1)”. Table 4 (for E2) shows violation of “ρt_0_*(t_1_) in It_0_*(t1)” in m = 1 of M = 6 cases (16.6%) while “ρT_A0_*(t_1_) in IT_A0_*(t1)” is violated in m = 3 of M = 6 cases (50%). The random variable m has a binomial distribution b(M, q, m) with the cumulative probability distribution B(M, q, m). We can interpret those results as the outcome of testing the null hypothesis H0 “Estimation errors distributions of t_0_* and of T_A0_* are Gaussians and Newtonian model sufficient for T_X_(t) and T_Y_(t)” using the data in Table 3 (E1) and Table 4 (E2). The critical range for α-level testing is: maximum CR_α_ := (m ≥ m_αL_) or (m_αU_ ≤ m) with B(M, q, CR_α_) ≤ α. For α = 0.05 the critical range for E1 (M = 16) is: CR_0.05_ = {0, …, 6} ᴜ {15, 16}, while we have for E2 (M = 6): CR_0.05_ = {1}. This means for E1: H0 is neither for t_0_* nor for T_A0_* rejected, and as well not for E2 with T_A0_*. But it is rejected for E2 in case of t_0_*. As the assumption of the estimators’ error distributions to be Gaussians should be either true or false for both experiments, the result indicates the Newtonian model to fit the data roughly, but not particularly well.

Our motivation to use the Newtonian model for a first approach is simplicity: it keeps the computations clear and concise. Note that our formulae are derived explicitly. Approaches using more complex models have to drop this condition.

Our result seems to represent a case of ‘underfitting’ for the Newtonian model in case of E1 at least: Despite optimum matching in the area used, the model is not able to correctly reconstruct the curve shape outside the input measurement interval.

Comparing relative errors ρt_0_^#^ and ρT_A0_^#^ in Tables 1 and 3 suggests that Newtonian cooling might be an overly simplistic model. Considering, e.g., for experiment E1:

T1 L1 $$\begin{array}{c}N=1,\;\triangle t\:=\:10min,t_1\:=\:5h\;50min,\;t_2=\:6h\;00min,\;\rho t_{0^\wedge}=-14\%,\;\rho T_{A0^\wedge}=5\%\end{array}$$

T3 L2 $$\begin{array}{c}N=100,\;\;\triangle t\:=10min,\;t_{1,1\;}=\;5h\;50min,\;t_{1,N}\;=\;8h\;19min,\;\rho t_{0\ast}=-24\%,\;\rho T_{A0\ast}=11\%\end{array}$$

shows a case where *N* = 100 leads to even slightly higher relative errors than *N* = 1. Examining the measured and the fitted curve with *N* = 100 shows a good fit in the interval [t_1,1,_ t_1,N_ + Δt] of the quadruple measurements, however, notable errors in t_0_* and T_A0_* are still present. This flaw is not resolved by enlarging the fitting interval [t_1,1,_ t_1,N_ + Δt] and increasing N accordingly. The following fitting result for E1 gives evidence to our statement, when compared to (T3 L2) above:

T3 L3 $$\begin{array}{c}N\:=\:250,\;\;\triangle t\:=\:10min,\;t_1\:=\:5h\;50min,\;t_{1,N}=\;9h\;59min,\;\rho t_{0\ast}=-18\%,\;\rho T_{A0\ast}=10\%\end{array}$$

Here, the relative errors decreased only slightly in comparison to (T3 L2) above. Again, the graphs of the measured and reconstructed curves T_X_(t), T_Y_(t), T_X_*(t), T_Y_*(t) present mostly correct matching in [t_1,1,_ t_1,N_ + Δt] but nevertheless the errors ρt_0_* and ρT_A0_* persist.

The measurements and computations presented are performed in a laboratory setting under maximum control of the relevant variables. In practical casework, additional challenges will arise, such as deviations of the ambient temperature curve T_A_(t) from the assumed piecewise constant shape, as observed in experiment E2. Even if direct comparison is difficult, the results indicate that the method might be robust to some extent because of similar orders of magnitude in the relative errors.

In experiment E2, significant deviations of the measured curve T_A_(t) from the two-plateau-scenario were involuntarily produced. Rather than discarding E2, we choose to proceed with it to test the robustness of our approach against such deviations. Comparing E1 and E2 directly is difficult because of different time ranges: [5 h 5 min, 10 h] in case of E1 and [2 h 38.5 min, 3 h 40 min] for E2.

Reconstructions with similar values of design parameters for E1 and E2 are e.g.:

T1 L1 $$\begin{array}{c}N=1,\;\triangle t\:=\:10min,t_1=5h\;50min=\:t_0+45min,\;t_2,\;\:6h\;00min,\;\rho t_{0^\wedge}=-14\%,\;\rho T_{A0^\wedge}=5\%\end{array}$$

T2 L6 $$\begin{array}{c}N=1,\;\triangle t=\:10min,\;t_1\:=\:3h\;20min\:t_0+42min,\;t_2=\:3h\;30min,\;\rho t_{0^\wedge}=10\%,\rho T_{A0^\wedge}=-13\end{array}$$

The relative errors are of the same order of magnitude roughly.

Another E1-E2-pair with similar design parameter values are:

T1 L9 $$\begin{array}{c}N=\:1,\;\triangle t\:=10min,\;t_1\:=\:6h\;40min\:t_0+95min,\;t_2\:=\:6h\;50min,\;\rho t_{0^\wedge}=-26\%,\;\rho T_{A0^\wedge}=17\%\end{array}$$

T2 L7 $$\begin{array}{c}N=1,\;\triangle t=10min,\;t_1=3h\;40min=t_0+65min,\;t_2=3h\;50min,\;\rho t_{0^\wedge}=-10\%,\;\rho T_{A0^\wedge}=-12\%\end{array}$$

Again, the relative errors exhibit a similar order of magnitude in both cases.

A more practical challenge is the positioning of the temperature probes for T_X_ and T_Y_ inside the boxes without opening them, which would destroy the temperature information about T_A0_ in the closed box quickly. Here, merely experimental methods may lead to results, such as drilling fine holes in cupboard or cardboard walls to insert the probes for T_Z_ measurements. It might even be necessary to introduce a thin optical fiber to explore the arrangement of the boxes’ content before implementing a temperature probe.

Further experiments and reconstructions are necessary to determine the limitations of our approach and to develop methods to address its problems, e.g., by using more complex cooling models for boxes in changing T_A_(t). In extreme cases, a finite element simulation of the cooling box could be used to handle strongly varying ambient temperatures. We consider the Newtonian cooling assumption as a first step which could be improved by more complex cooling models. Especially for short time distances t_1_ - t_0_, we believe that deviations of t_0_ and T_A0_ estimates and of the reconstructed cooling curves (see Fig. 3) could be diminished. Moreover, the quadruple measurement distance Δt needs to be chosen carefully. While choosing Δt too small increases susceptibility to measurement errors, a too large value of Δt moves the second measurement into the asymptotic regime of T_Z_(t) converging to T_A1_. The corresponding loss of information would again lead to strongly increasing estimation errors in t_0_^ and T_A0_^. Consequently, it would make sense to bound the time interval, where quadruples can be advantageously used for t_0_* and T_A0_*, which may be called localization.

The impact of variations in T_A0_ and t_0_ estimation on TTDE results can be quantified, in principle, (see e.g. [1]. for FEM-TTDE) if the TTDE method allows for changing T_A_. Here, the t_0_-error δt_0_ plays a special role since it simply shifts the correct TDE-result t_D_^+^ to t_D_^+^ + δt_0_. Even errors of high relative values of 10% or 20% seem to be tolerable if the value of δt_0_ stays smaller than the standard deviation of the TDE result. However, a valid estimation of the resulting error in TDE from t_0_* and T_A0_* error is a desideratum. Resulting errors for TDE can be estimated via Gaussian error propagation or Monte Carlo methods as in [1, 2].

In cases of large temperature differences T_A0_ – T_A1_, the usual tolerance interval of MHH is insufficient to handle the resulting TTDE error [2], which prevents approximate solutions with estimated constant mean ambient temperatures. To address this problem, Althaus and Henßge [4] attempted to adapt the MHH model with instantaneous ambient temperature changes but assumed known values for t_0_ and T_A0_. Their approach used a sequential twofold MHH application with an altered MHH model for the first step. The authors fitted the parameter A’s value for their model using experimental dummy cooling data in a scenario with an instantaneous temperature drop from T_A0_ down to T_A1_. Said fitting result for A therefore depends on the temperatures T_A0_ and T_A1_. An error estimation for the resulting TDE could thus be performed via Monte Carlo Simulation in a laborious process.

## Conclusion

While our results demonstrate the potential of the method for t_0_ and T_A0_ reconstruction in principle and suggest its applicability in case work, particularly when the temperature drop occurred recently and moderate variations in TDE are acceptable, further research is needed to explore more elaborate models, estimation techniques, or localization algorithms to enhance the method’s accuracy. Moreover, an estimation of the (t_0_, T_A0_)-reconstruction error’s impact on the final TDE error is essential.

## Data Availability

The program code is available from the corresponding author on reasonable request.
